# Naturally inspired SERS substrates fabricated by photocatalytically depositing silver nanoparticles on cicada wings

**DOI:** 10.1186/1556-276X-9-298

**Published:** 2014-06-12

**Authors:** Ichiro Tanahashi, Yoshiyuki Harada

**Affiliations:** 1 Nanomaterials and Microdevices Research Center, Osaka Institute of Technology, 5-16-1 Omiya, Asahi-ku, Osaka 535-8585, Japan

**Keywords:** Silver nanoparticles, SERS, Localized surface plasmon resonance, Cicada, Wing

## Abstract

Densely stacked Ag nanoparticles with an average diameter of 199 nm were effectively deposited on TiO_2_-coated cicada wings (Ag/TiO_2_-coated wings) from a water-ethanol solution of AgNO_3_ using ultraviolet light irradiation at room temperature. It was seen that the surfaces of bare cicada wings contained nanopillar array structures. In the optical absorption spectra of the Ag/TiO_2_-coated wings, the absorption peak due to the localized surface plasmon resonance (LSPR) of Ag nanoparticles was observed at 440 nm. Strong Surface-enhanced Raman scattering (SERS) signals of Rhodamine 6G adsorbed on the Ag/TiO_2_-coated wings were clearly observed using the 514.5-nm line of an Ar^+^ laser. The Ag/TiO_2_-coated wings can be a promising candidate for naturally inspired SERS substrates.

## Background

Noble metal nanoparticles with localized surface plasmon resonance (LSPR) absorption in the visible wavelength region have a wide variety of beautiful colors. These noble metal nanoparticles have been applied in the field of nonlinear optics [1,2], biological and chemical sensing, and surface-enhanced Raman scattering (SERS) [3,4]. Among the noble metal nanoparticles, silver (Ag) and gold (Au) nanoparticles are one of the most investigated SERS-active metal nanoparticles because of their clear LSPR absorption [5-8].

In recent years, a lot of studies have been carried out focusing on the preparation of SERS-active substrates with larger area, low cost, and high performance [3-14]. The LSPR of a noble metal nanoparticle is primarily responsible for the SERS effect [5-8] and the LSPR properties are strongly dependent on the size and shape of the nanoparticles. The surface nanostructures of the substrates affect the properties of the nanoparticles deposited on the substrates. New types of SERS-active substrates have been developed by using the nanostructures of butterfly and cicada wings [9-14]. It is known that the butterfly and cicada wings have a number of predominant optical effects such as antireflection and photonic bandgap [15,16]. Especially, the wings of some kinds of cicadas have nanopillar array structures and they show excellent antireflection properties. Usually, nanopillar array structures with tunable gap size are fabricated by electron-beam lithography [11]. On the other hand, the cicada wings composed of chitin are a self-assembled natural nanocomposite material.

In our previous studies [17,18], we have reported that the photocatalytically prepared Ag and Au nanoparticles deposited on TiO_2_ films showed the excellent SPR-sensing properties. Photocatalytic deposition method seems to be a convenient and desirable method to obtain stable and immobilized metal nanoparticles on the substrates. Thus, we have applied the photocatalytic deposition method to fabricate Ag nanoparticles deposited on the cicada wings with nanopillar array structures as SERS-active substrates.

In this paper, we have reported the preparation and SERS properties of the Ag nanoparticles deposited on TiO_2_-coated cicada wings with uniformly ordered nanopillar array structures.

## Methods

### The preparation of Ag/TiO_2_-coated wings, Ag/wings and Ag films

The preparation processes of the Ag nanoparticles deposited on TiO_2_-coated cicada wings (Ag/TiO_2_-coated wings) and Ag nanoparticles deposited on cicada wings (Ag/wings) without TiO_2_ are outlined as follows. Cicada wing samples were collected from a Japanese endemic species *Cryptotympana facialis* (a black cicada with clear and transparent wings). The cicadas were captured locally in Osaka City, Japan. In this experiment, the dorsal forewings of male cicadas were used. Before the dip-coating process, the forewings (50 to 55 mm in length) of individual cicada were rinsed using ethyl alcohol and deionized water to remove contaminant and dried at room temperature. TiO_2_ was coated on both sides of the forewing from anatase sol (Ishihara Sangyo Kaisha, ST-K211) by using a dip-coating technique. The resulting wing was soaked in a mixture of 2 mL of a 5.0 × 10^-2^ mol L^-1^ AgNO_3_ aqueous solution and 4 mL of ethyl alcohol (1.67 × 10^-2^ mol L^-1^ of Ag^+^ ions) in a petri dish (5 cm in diameter) about 10 mm away under a 15-W low-pressure mercury lamp (a germicidal lamp) with a power density of 0.13 mWcm^-2^ for 1 h. In this process, Ag^+^ ions were photoreduced on the surface of TiO_2_. Forewings without TiO_2_ were also treated as the abovementioned procedure. Ag^+^ ions were also photoreduced on the surface of the cicada wings (chitin) without TiO_2_ (Ag/wings). The resultant Ag/TiO_2_-coated wings and Ag/wings were washed with deionized water, finally dried in air. All the preparation procedures were carried out at room temperature. As a reference, Ag films deposited on a glass slide were prepared by a magnetron sputtering system. The Ag (99.9%, 2 in. in diameter) target was used. Sputtering was carried out in Ar gas of 1 to 2 Pa and the applied power of the Ag target was 50 W. The glass slide substrates were not intentionally heated during the sputtering. All compounds were of reagent grade and were used without further purification.

### The XRD and SEM measurements

X-ray diffraction (XRD) measurements were performed on a RINT 2000 X-ray diffractometer (Rigaku Corporation, Tokyo, Japan), using Cu Kα radiation working at 40 kV and 40 mA. The crystallite size, *d*, of the samples was estimated using the Scherrer equation: *d* = 0.9*λ*/*β*cos*θ*, where *λ* is the wavelength of X-ray source (0.154059 nm) and *β* is the full width at half maximum (FWHM) of the X-ray diffraction peak at the diffraction angle *θ*. Scanning electron microscopy (SEM) analysis of the bare cicada wings, Ag/wings, Ag/TiO_2_-coated wings and Ag films was carried out using a VE-8800 scanning electron microscope (Keyence Corporation, Osaka, Japan) at an acceleration voltage of 15 kV and a working distance of 4 to 12 mm.

### The UV–Vis absorption spectra and SERS spectra measurements

All absorption spectra were recorded from 200 to 800 nm on an UV-3100PC dual beam spectrophotometer (Shimadzu Corporation, Kyoto, Japan). For SERS measurements, the sample was irradiated with 50 mW of 514.5-nm line (Ar^+^ laser) in back scattering geometry at room temperature. A × 50-long distance objective and a cooled CCD detector were employed. The laser beam was focused on a spot with a diameter of approximately 2 μm and the data acquisition time for each measurement was 1 s. Optical images were obtained with the camera attached to the Raman microscope. The Raman spectra of 10^-3^ mol L^-1^ Rhodamine 6G (R6G, 2 μL) adsorbed on various samples were compared. For the bare cicada wings, Ag/wings, and Ag/TiO_2_-coated wings, R6G was adsorbed on the surface at the center of the cicada dorsal forewings.

## Results and discussion

### Colors and SEM micrographs of the bare cicada wings, Ag/wings, Ag/TiO_2_-coated wings and Ag films

In the case of the Ag/wings, the color of bare cicada wings was changed from clear transparent to dark brown after the photoreduction of Ag^+^ ions onto the wings. On the other hand, the color of the wings was changed from clear transparent to metallic gray for the case of the Ag/TiO_2_-coated wings. These color changes indicated the formation of Ag metal on the wings. Photoreduction of Ag^+^ ions on TiO_2_-coated wings was faster than that on the wings without coated TiO_2_. This is due to that the coated TiO_2_ works as a photocatalyst effectively. On the other hand, the color of the Ag film prepared by the sputtering was metallic silver.

**Figure 1:**
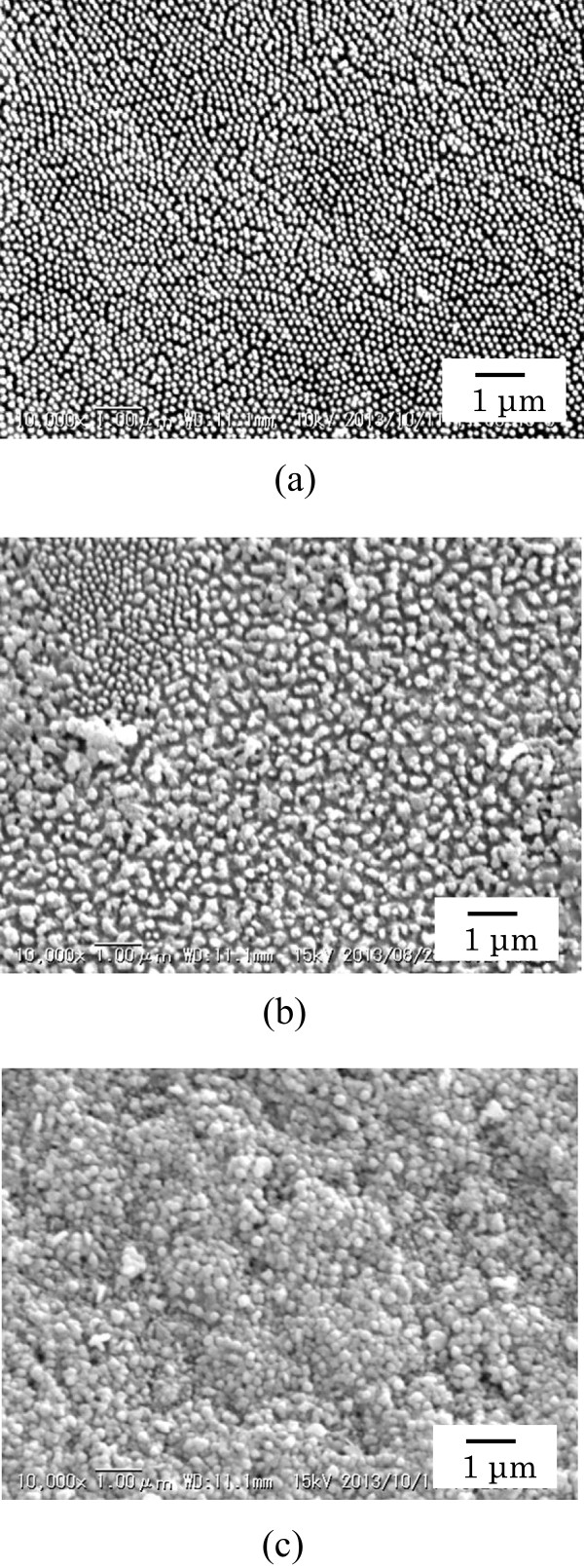
**SEM micrographs of the (a) bare cicada wing, (b) Ag/wing, and (c) Ag/TiO**_**2**_-**coated wing.**

Typical SEM image of the dorsal forewing of male cicada (*Cryptotympana facialis*) is shown in Figure 1a. In the figure, a dense nanopillar array structure with a large area is seen. Diameters and separations of the array of nanopillars are about 130 and 30 to 130 nm, respectively. From other SEM images not shown here, the nanopillar was found to be about 300 nm in height. The morphology of the surface structures was almost the same for the dorsal and ventral surfaces and between male and female specimens. It has been suggested that these structures have an antireflection property [15]. Figure 1b,c shows SEM images of the Ag/wing and Ag/TiO_2_-coated wing, respectively. In Figure 1b, it is seen that a part of surface is covered with irregular-shaped Ag particles. In the photoreduction process, it seems that Ag^+^ ions are not uniformly reduced on the functional groups of chitin of the wings. On the other hand, densely stacked Ag nanoparticles are seen in Figure 1c. A part of the micrograph field including 150 particles was randomly selected to analyze the size distribution. The average diameter of the nanoparticles was estimated to be 199 nm with a standard deviation of 41 nm. The size of the Ag nanoparticles on TiO_2_-coated wings was larger than that of Ag nanoparticles (113 nm) on TiO_2_-coated glass slides [17]. It is thus that the densely stacked Ag nanoparticles with 199 nm in average diameter were successfully prepared on TiO_2_-coated three-dimensional nanopillar array structures of the cicada wings. On the other hand, in the SEM images of the Ag film not shown here, the surface was smooth and the nanoparticles and nanopillars were not seen in the images.

### XRD patterns of the bare cicada wings, Ag/wings, Ag/TiO_2_-coated wings and Ag films

**Figure 2:**
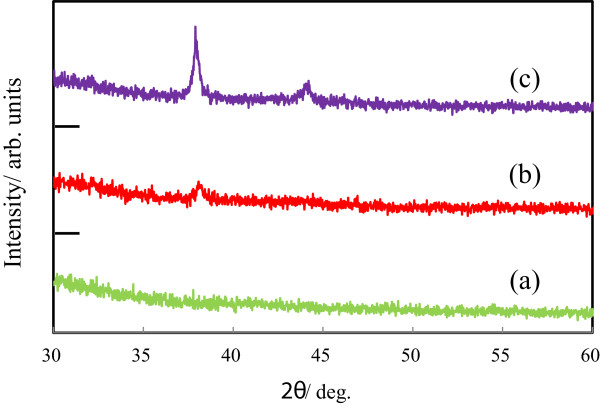
**X-ray diffraction patterns of the (a) bare cicada wing, (b) Ag/wing, and (c) Ag/TiO**_**2**_-**coated wing.**

Figure 2 shows the XRD patterns of the (a) bare cicada wing, (b) Ag/wing, and (c) Ag/TiO_2_-coated wing. In the figure, no distinct diffraction peaks is seen for the (a) bare cicada wing. On the other hand, both the (b) Ag/wing and (c) Ag/TiO_2_-coated wing show the peak at 2*θ* = 38.1° which was assigned to the (111) reflection lines of cubic Ag. Together with the peak at 2*θ* = 38.1°, the peak at 2*θ* = 44.3°, (200) reflection lines of cubic Ag, is also observed in the patterns of the (c) Ag/TiO_2_-coated wing. Therefore, the amount of deposited Ag for the Ag/TiO_2_-coated wing seems to be larger than that of the Ag/wing. The crystallinity of the Ag for the Ag/TiO_2_-coated wing also seems to be higher than that of the Ag/wing. In the XRD patterns of the Ag film, the peaks at 2*θ* = 38.1° and 44.3° were also clearly seen and the crystallite size of Ag was calculated to be 19.6 nm from the peak (2*θ* = 38.1°) broadening. On the other hand, the crystallite sizes of Ag nanoparticles deposited on the Ag/wing and Ag/TiO_2_-coated wing were 12.7 and 22.0 nm, respectively. Therefore, the Ag nanoparticles and Ag film were consisting of small Ag crystallites and the crystallite sizes of Ag nanoparticles deposited on the bare wing and TiO_2_-coated wing and the Ag films were almost the same.

### UV–Vis absorption spectra of the bare cicada wings, Ag/wings, and Ag/TiO_2_-coated wings

**Figure 3:**
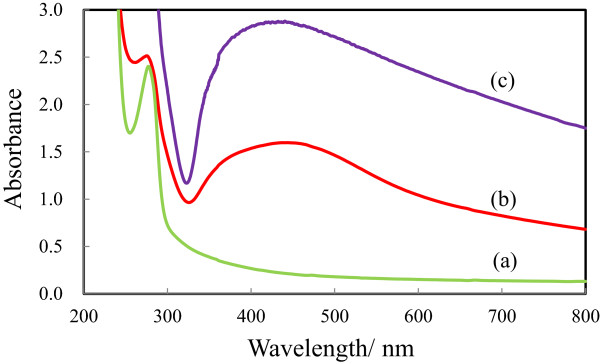
**Optical absorption spectra of the (a) bare cicada wing, (b) Ag/wing, and (c) Ag/TiO**_**2**_-**coated wing.**

Figure 3 shows the absorption spectra of the (a) bare cicada wing, (b) Ag/wing, and (c) Ag/TiO_2_-coated wing. In the figure, an absorption peak at 275 nm, due to the nanopillar array structure on the cicada wings is seen for the (a) bare cicada wing [9]. In Figure 3, the (b) Ag/wing and (c) Ag/TiO_2_-coated wing show the broad LSPR absorption band of the Ag nanoparticles peaking at about 440 nm. The broad absorption bands of the (b) Ag/wing and (c) Ag/TiO_2_-coated wing suggest that the shape variation and the size distribution of the Ag nanoparticles are large. In both the spectra, the broad absorption band at a longer wavelength than that of the LSPR peak is probably due to the light scattering of the larger size Ag nanoparticles of the (b) Ag/wing and (c) Ag/TiO_2_-coated wing.

### SERS spectra of R6G adsorbed on the surface of the bare cicada wings, Ag/wings, Ag/TiO_2_-coated wings and Ag films

**Figure 4:**
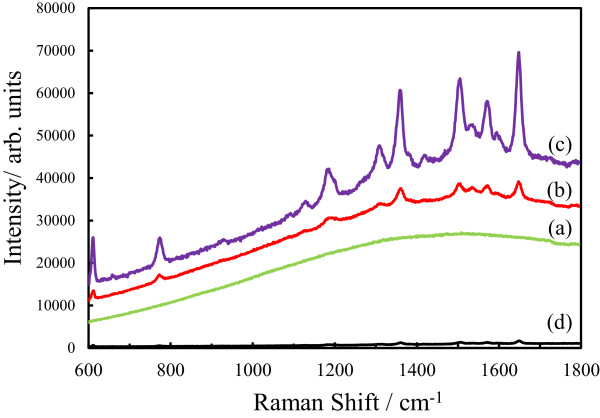
**SERS spectra.** R6G adsorbed on the (a) bare cicada wing, (b) Ag/wing, (c) Ag/TiO_2_**-**coated wing, and (d) Ag film on a glass slide.

SERS spectra of R6G adsorbed on the (a) bare cicada wing, (b) Ag/wing, and (c) Ag/TiO_2_-coated wing are shown in Figure 4. In this SERS measurement, R6G as standard remarks was adsorbed on the surface at the center of the dorsal forewings with an area of about 54 mm^2^. The SERS spectrum of R6G adsorbed on the (d) Ag film deposited on a glass slide prepared by sputtering is also shown in Figure 4. In the case of the (d) Ag film, the R6G-adsorbed area was about 50 mm^2^ which was almost the same as those of the (a) bare cicada wing, (b) Ag/wing, and (c) Ag/TiO_2_-coated wing. In the figure, R6G adsorbed on the (a) bare cicada wing shows no distinct peaks. A broad band of the spectrum from 600 to 1,800 cm^-1^ was due to the photoluminescence of R6G. On the other hand, R6G adsorbed on the (b) Ag/wing, (c) Ag/TiO_2_-coated wing, and (d) Ag film show the distinct SERS peaks. In Figure 4, the observed Raman bands seen in the (b) Ag/wing, (c) Ag/TiO_2_-coated wing, and (d) Ag film are assigned to R6G include ν(C-H) out-of-plane bend mode at ca. 774 cm^-1^, ν(C-H) in-plane bend mode at ca. 1,129 cm^-1^, ν(C-C) stretching mode at ca. 1,358, 1,505, and 1,649 cm^-1^[7,19]. The peak intensities of R6G adsorbed on the (a) bare cicada wing, (d) Ag film, (b) Ag/wing, and (c) Ag/TiO_2_-coated wing became large in that order. The peak intensity of R6G at 1,649 cm^-1^ of the (c) Ag/TiO_2_-coated wing was 36 times larger than that of the (d) Ag film and it was 6 times larger than that of the (b) Ag/wing. From the results of SEM and XRD of the bare cicada wings, Ag/wings, Ag/TiO_2_-coated wings, and Ag films, SERS properties of these samples are mainly influenced by the nanostructures of their surfaces.

## Conclusions

By using the self-assembled natural nanopillar array structures of the cicada wings and TiO_2_ photocatalyst, SERS-active substrates of the Ag/TiO_2_-coated wings with larger area, low cost, and high performance were successfully prepared. Densely stacked Ag nanoparticles with 199 nm in average diameter were easily and effectively deposited on the TiO_2_-coated cicada wings. In the optical absorption spectra of the Ag/TiO_2_-coated wings, the absorption peak due to the LSPR of Ag nanoparticles was observed at 440 nm. In the SERS spectra (514.5 nm excitation line), the peak intensity of R6G at 1,649 cm^-1^ of the Ag/TiO_2_-coated wing was 36 times larger than that of the Ag film. The Ag/TiO_2_-coated wings can be used as SERS substrates.

## Abbreviations

LSPR: localized surface plasmon resonance; SERS: surface-enhanced Raman scattering; XRD: X-ray diffraction; SEM: scanning electron microscope.

## Competing interests

The authors declare that they have no competing interests.

## Authors’ contributions

IT conceived of this study; carried out the preparation of the SERS substrates, the Ag/TiO_2_-coated wings, Ag/wings, and Ag films; performed the XRD, SEM, and optical absorption measurements; and drafted the manuscript. YH performed the SERS measurements. Both authors read and approved the final manuscript.
